# Opsoclonus: A Rare Neurological Manifestation in a Patient With Scrub Typhus Infection

**DOI:** 10.7759/cureus.70058

**Published:** 2024-09-23

**Authors:** Akhil Neela, Rucha Gohil, Ravindra Tagore, Vidya TA

**Affiliations:** 1 General Medicine, SRM Medical College Hospital and Research Centre, Chengalpattu, IND

**Keywords:** neurological manifestations, neurology, nystagmus, opsoclonus, scrub typhus, tropical infections

## Abstract

Scrub typhus, prevalent in tropical regions, exhibits a wide range of symptoms, from non-specific signs to severe conditions such as pneumonia, gastroenteritis, lymphadenitis, meningitis, encephalitis, acute kidney injury, and multi-organ dysfunction syndrome. Neurological symptoms like opsoclonus are rarely seen. This report details an unusual case of a 34-year-old male who first complained of high temperature, headache, and sore muscles. Initially treated with antipyretics and oral antibiotics, his symptoms persisted and new ones emerged, leading to an emergency visit with complaints of blurred vision. Upon confirming scrub typhus with opsoclonus, appropriate antibiotics were administered. Persistent fever and opsoclonus prompted suspicion of an atypical infection. A detailed history and investigations, including IgM testing against the scrub typhus antigen, confirmed the diagnosis. Treatment with doxycycline resulted in significant symptom improvement, leading to discharge. This case underscores the need to consider atypical organisms in neurological symptoms, which can be effectively treated with timely diagnosis.

## Introduction

Scrub typhus is an acute, febrile, infectious illness caused by *Orientia tsutsugamushi*. *Orientia tsutsugamushi*, belonging to the Rickettsiaceae family, is the causative agent of scrub typhus, sometimes referred to as tsutsugamushi disease. It is transmitted through the bites of infected chiggers and is endemic in rural Asia, the western Pacific, and northern Australia. Prompt diagnosis and antibiotic treatment are essential because the disease can sometimes be fatal. The disease often presents with fever, headache, muscle pain, and a distinctive eschar at the bite site but can also lead to severe complications like pneumonia, meningitis, encephalitis, lymphadenitis, gastroenteritis, hepatitis, acute kidney injury, and multi-organ dysfunction. Without treatment, the disease can be fatal, with mortality influenced by strain virulence, patient characteristics, diagnostic delays, and drug resistance.

Scrub typhus is sometimes associated with neurological manifestations, among which opsoclonus is one of the rare findings. Opsoclonus, a rare neurological symptom, is caused by various underlying conditions, most often paraneoplastic or para-infectious. It is caused by brain-stem dysfunction with accompanying cerebellar and/or cerebellar pathway dysfunction, as suggested by a few radiographic and pathological examinations. The pathophysiology of this illness involves immunologic and/or inflammatory mechanisms [1]. It is critical to identify this syndrome as soon as possible. Diagnosing scrub typhus in patients with opsoclonus requires high clinical suspicion, confirmed by polymerase chain reaction and indirect immunofluorescence assay, with a significant rise in IgM antibodies indicating infection.

## Case presentation

A 34-year-old male presented to the emergency department with a 10-day history of intermittent high fever, headache, and muscle pain, later developing blurred vision. His family noticed abnormal, non-specific eye movements, especially when he tried to focus on objects. He had no difficulty in speech, swallowing, hearing, limb weakness, sensory disturbances, altered sensorium, or bowel or bladder issues. His medical, family, and drug histories were unremarkable.

Upon assessment, he had a fever, a pulse of 110 beats per minute, blood pressure of 110/70 mmHg, and a respiratory rate of 18/min. He exhibited no pallor, icterus, cyanosis, clubbing, lymphadenopathy, or rashes. Neurologically, he was conscious and oriented, with a Glasgow Coma Scale score of 15/15, and no sensorimotor or cerebellar deficits. On examination, no cranial nerve involvement was found, but an eye examination revealed multiaxial, involuntary saccadic movements indicative of opsoclonus without limitations of involuntary eye movements (Video 1).

**Video 1:** Opsoclonus: bilateral multiaxial, involuntary saccadic movements.

**Figure 1:**
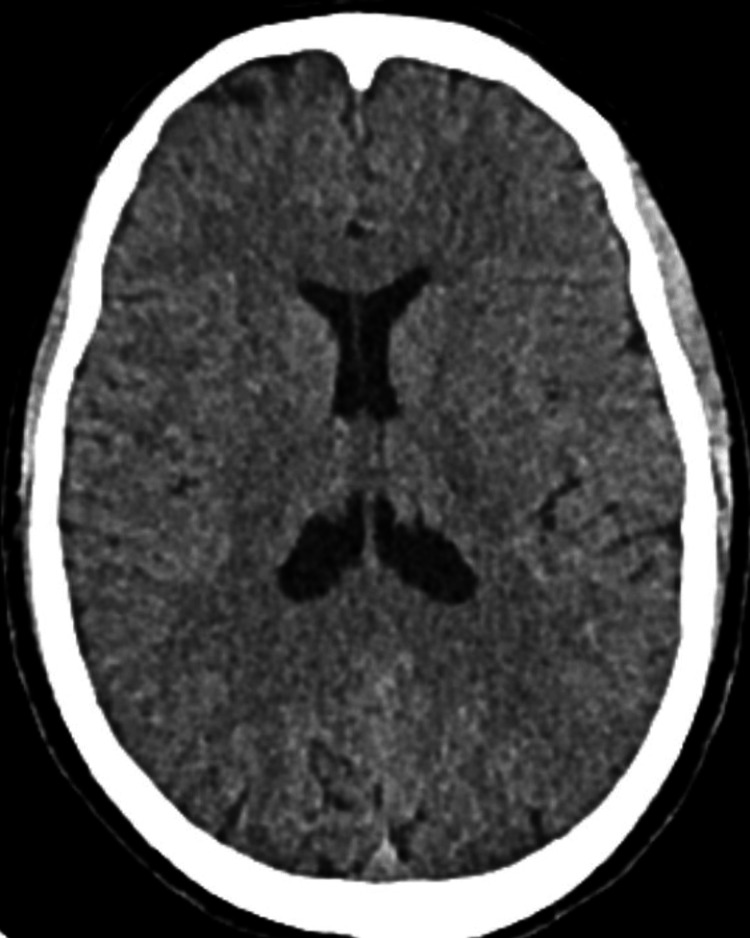
Non-contrast CT of the brain shows no significant abnormalities.

**Figure 2:**
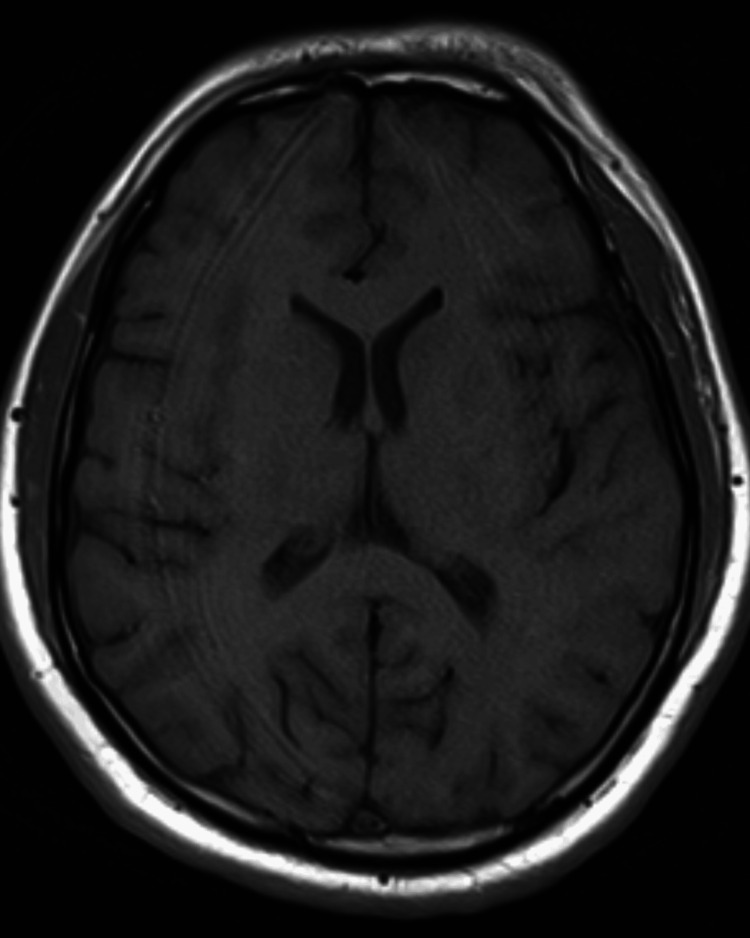
Axial T1-weighted MRI of the brain showing normal findings.

Laboratory tests revealed an increased total leukocyte count with lymphocytosis, normal hemoglobin, platelets, erythrocyte sedimentation rate, random blood sugar, serum urea, and creatinine levels (Table 1). Blood cultures were sterile, and rapid diagnostic tests for malaria were negative. IgM ELISA for dengue, scrub typhus, and Leptospira was performed. Imaging (non-contrast CT and MRI) of the brain was found to be normal (Figures 1-2).

**Table 1:** Serial monitoring of hematological and biochemical parameters. Hb: Hemoglobin; TLC: Total leukocyte count; mEq/L: Milliequivalents per liter; RBS: Random blood sugar.

Parameters	Day 1	Day 3	Day 5	Day 10	Reference range
Hb (%)	13.7	13.3	13.5	13.1	13-16
TLC (x 1000)	15.5	14.2	9.9	6.7	5-11
Platelet Count (x 1000)	245	250	262	247	150-420
Serum Urea (mg/dL)	27	25	20	25	15-30
Creatinine (mg/dL)	0.7	0.7	0.8	0.9	0.5-1.0
Sodium (mEq/L)	136	138	130	136	130-145
Potassium (mEq/L)	4.1	4.3	4	4.3	3.5-5.0
Albumin (mg/dL)	3.8	3.5	3.6	3.6	3.0-4.0
RBS (mg/dL)	117	154	134	164	<200

CSF analysis showed mild elevation of protein and mild lymphocytic pleocytosis. His IgM titer for scrub typhus was positive (1:3200), while his IgM for dengue and Leptospira was negative (Table 2).

**Table 2:** Cerebrospinal fluid analysis. CSF: Cerebrospinal fluid; Polys: Polymorphonuclear cells.

Lab test - CSF analysis	Lab values	Reference values
Color	Colorless	Colorless
Appearance	Clear	Clear
Glucose level (mg/dL)	47	40-70
Protein (mg/dL)	71	15-60
WBC (cells/mm3)	11	0-5
RBC (cells/mm3)­­­­	0	0-5
Polys CSF (%)	72	NA
Lymphocytes CSF (%)	2	NA
Monocytes CSF (%)	8	NA
CSF culture	No growth after 72 hours	NA

The diagnosis of scrub typhus with opsoclonus was confirmed. He was treated with intravenous doxycycline 100 mg twice daily and azithromycin. The patient showed rapid improvement, with opsoclonus decreasing after three days and resolving completely within four days of treatment.

## Discussion

Scrub typhus, a major public health concern, is caused by the rickettsial bacteria *Orientia tsutsugamushi*, which is frequently found in the Tsutsugamushi Triangle. Its incidence is 4.6 per 100,000 over 10 years, with a 13.6% case fatality rate [2]. Systemic manifestations of the disease may begin to develop in the second week, ranging from central nervous system manifestations such as meningitis, encephalomyelitis, encephalopathy, and sometimes cranial nerve palsies and ocular involvement [3-5].

Scrub typhus rarely manifests as opsoclonus, an uncommon neurological condition that typically coexists with myoclonus, cerebellar impairment, or extrapyramidal symptoms. The neurological abnormalities appear to fully resolve during the febrile period. Opsoclonus is caused by the breakdown of Purkinje cells in the dorsal part of the vermis, which disrupts the inhibitory tone of saccadic burst neurons in the pontine reticular formation and leads to disinhibition of the cerebellar fastigial nucleus [6,7].

In general, opsoclonus can be attributed to parainfectious, paraneoplastic, and other causes. It is more common in the young population, where neuroblastoma is the most frequent cancer, and paraneoplastic diseases predominate over parainfectious presentations. Information regarding opsoclonus in the adult population is limited; the probability of presentations being due to parainfectious causes and paraneoplastic conditions is equal; thus, paraneoplastic conditions need to be ruled out [8,9].

Lyme disease, streptococcal infection, varicella zoster virus [10], Epstein-Barr virus, and Coxsackie B virus are among the infections associated with opsoclonus. According to a few reports, patients who were febrile and exhibited opsoclonus during examination had an infection with scrub typhus, which responded to treatment with doxycycline [11].

Our patient showed no signs or indicators of malignancy. No further assessment was carried out because the opsoclonus quickly resolved with treatment for scrub typhus. As far as we are aware, this is just one of the few reports that have been published on this unusual presentation of scrub typhus. It emphasizes how uncommon presentations can occur even in common and well-known diseases.

## Conclusions

Scrub typhus can cause opsoclonus, a rare yet important neurological symptom. For successful outcomes, early diagnosis and appropriate antibiotic therapy are essential. This case report emphasizes the importance of ruling out scrub typhus in patients exhibiting neurological symptoms, particularly in areas where the disease is endemic, in order to avoid serious consequences and ensure a prompt recovery.
